# COVID-19 Increases the Risk of New Myocardial Infarction in Patients with Old Myocardial Infarction: A Retrospective Observational Study

**DOI:** 10.1177/11795468241301133

**Published:** 2024-12-18

**Authors:** Ayman El-Menyar, Ahmed Faidh Ramzee, Mohammad Asim, Fakhar Shahid, Yaser M Ata, Hamzah El Baba, Areen Fino, Arun P Nair, Ruben Peralta, Muna A Almaslamani, Jassim Al Suwaidi, Hassan Al-Thani, Sandro Rizoli

**Affiliations:** 1 Clinical Research, Trauma and Vascular Surgery, Hamad Medical Corporation, Doha, Qatar; 2 Clinical Medicine, Weill Cornell Medical College, Doha, Qatar; 3 Trauma Surgery Section, Hamad Medical Corporation, Doha, Qatar; 4 Department of Surgery, Hamad Medical Corporation, Doha, Qatar; 5 Department of Family Medicine, Hamad Medical Corporation, Doha, Qatar; 6 Communicable Disease Center (CDC), Hamad Medical Corporation, Doha, Qatar; 7 Department of Surgery, Universidad Nacional Pedro Henriquez Urena, Santo Domingo, Dominican Republic; 8 Department of Cardiology, Heart Hospital, Hamad Medical Corporation, Doha, Qatar

**Keywords:** Myocardial infarction, thromboembolic events, COVID-19, cytokines, ACE-2 mediated, SARS-CoV-2

## Abstract

**Background:**

We aimed to investigate the incidence of new acute myocardial infarction (AMI), in patients with Coronavirus disease (COVID-19) who had old MI. We hypothesized that COVID-19 increases the rate of repeated AMI in this population regardless of age and gender.

**Methods:**

A retrospective analysis was conducted for adult patients admitted with COVID-19 and developed thromboembolic event (TEE) in 2020. Patients were categorized based on the history of old MI, new MI, age, and gender.

**Results:**

Among 16,903 patients with COVID-19 who were admitted, 210 (1.2%) developed TEE (89% were males, 55% were <55 years old, and 80.5% had an old MI). COVID-19 was severe in 32% of cases. AMI occurred in 160 patients (42.5% STEMI and 57.5% NSTEMI). In patients with prior MI, 92.5% developed another AMI. NSTEMI was higher in patients with severe COVID-19 than STEMI (33% vs 21%). Patients with severe COVID-19 had higher mortality (39.4% vs 5.6%), fewer rates of prior MI (74% vs 83%), hypertension (40% vs 60%), and STEMI (31.8% vs 46.5%) than mild COVID-19 patients. On multivariable analysis, COVID-19 severity was an independent predictor of mortality (OR10; 95%CI 1.62-67.19) after adjustment for age, gender, diabetes mellitus, C-reactive protein, serum Ferritin, Procalcitonin, and Fibrinogen values, and prior or new MI.

**Conclusions:**

Patients with old MI could develop a new AMI in 80% of COVID-19. However, the mortality was higher in patients without a history of MI due to the severity of COVID-19. Attention should be given to patients who possess thrombotic risk factors in pandemics.

## Introduction

Initially, the Coronavirus disease (COVID-19) was primarily considered a respiratory disease; however, recent evidence suggests that the severe acute respiratory syndrome coronavirus-2 (SARS-CoV-2) infection has complex clinical manifestations that may affect the multiorgan system.^1^ It has been observed that the coagulation process in individuals with COVID-19 is markedly deranged compared to healthy individuals.^2^ Many publications have reported a remarkably higher incidence of thromboembolic events (TEE) in patients with severe COVID-19, indicating that the viral infection might play a role in the hypercoagulable state.^3-6^ Notably, there is a diverse range of incidences of TEE in the literature, as some studies have documented relatively lower TEE frequencies ranging between 20% and 30%,^4,7,8^ while others have reported considerably higher rates of TEE (40%-70%).^3,9,10^ The coagulation derangement has been attributed to the hyperinflammatory response, cytokine storm, and increased platelet activation induced by viral particles, which enter the blood circulation by targeting endothelial ACE-2 receptors. SARS-CoV-2, by binding the ACE-2 receptors expressed on the host cell surface, leading to ACE-2 downregulation, with subsequent hyperactivation of the Ang II/AT1 system, cause vasoconstrictive, proinflammatory, and prooxidant effects.^5,11-13^ Also, COVID-19 promotes release of IL-1, IL-6, IL-7, TNFα, and IFNγ. These Cytokines can potentially impair endothelial function with increased production of oxidative stress and prothrombotic factors.^12,13^ Patients with COVID-19 may have higher levels of D-dimer, fibrinogen, and factor VIII, and many thromboembolic manifestations are observed in the lungs of critically ill patients on post-mortem examination.^1^

However, the pathology of coagulation defects in patients with COVID-19 is not fully understood. It is believed to differ from other coagulopathic disorders associated with critical illness, such as disseminated intravascular coagulopathy (DIC) and sepsis-induced coagulopathy (SIC), and it is labeled as COVID-19-associated coagulopathy (CAC).^14^ Prophylactic anticoagulation, regardless of thromboembolism risk assessment, is considered for the management of severe cases of COVID-19 based on expert opinion, shared experiences, and published best practices.^15^ Various international guidelines recommended the use of a universal thromboprophylaxis regime for COVID-19 patients.^16^ Additionally, the use of biomarkers such as D-dimer to adjust the dose of anticoagulants for thromboprophylaxis is no longer recommended due to insufficient data.^15^ Type 1 and II acute myocardial infarction (AMI) during COVID-19 is attributed to many factors such as direct viral cellular damage, systemic inflammatory response with cytokine-mediated injury, microvascular thrombosis, endothelial dysfunction, oxygen supply/demand imbalance, promoted atherosclerotic plaque instability and thrombus formation, enhances platelets activation and aggregation and upregulates the sympathetic nervous system resulting in increased vaso-motility and coronary spasm.^12,13^ Recent publications have found a significantly increased risk of myocardial infarction (MI) in patients with COVID-19.^17-19^ A growing number of reports highlighted the association between cardiovascular symptoms and thrombotic complications that occur in COVID-19 patients.^20^ However, it remains unclear if the increased thromboembolic risk or the burden of severe acute illness triggers AMI in COVID-19 patients. This study aimed to investigate the incidence and type of TEE, particularly AMI, in patients hospitalized with COVID-19 with an old MI. We hypothesized that COVID-19 increases the rate of new TEE in terms of an MI in this population regardless of age and gender.

## Methods

This retrospective study was conducted for all adult patients admitted for treatment in quarantine centers in Qatar with COVID-19 and developed TEE during the hospital course. In Qatar, the COVID-19 pandemic management is coordinated by the Hamad Medical Corporation (HMC), under the purview of the Ministry of Public Health, with uniform standards and protocols applied to patients across all hospitals. Therefore, nationally representative data obtained from the electronic medical records (CERNER) at Hamad Medical Corporation (HMC) allowed us to capture potentially all patients with clinically significant TEE and COVID-19 data between 17 February and 31 August 2020. Primarily, four hospitals were designated as COVID-19 treatment facilities, namely Hazm Mebaireek General Hospital, Communicable Disease Center, Mesaieed Hospital, and Ras Laffan Hospital). All quarantine centers were managed under the Communicable Disease Center. During the first wave of COVID-19 (2019-2020), all symptomatic patients were admitted to hospitals managed by the central health system. Asymptomatic patients were placed in the quarantine facilities per the Qatar Ministry of Public Health policy. This ensured that all patients with COVID-19 across the nation were monitored and managed by a unified healthcare entity following a consistent standard of care provided to all the patients.

The study included all laboratory-confirmed SARS-CoV-2 infection patients who were 18 years and above admitted to the hospital or quarantine facility. Patients aged below 18 years or lacking information regarding the TEE were excluded from the study.

Patients’ electronic health records were reviewed to retrieve data regarding demographics (age and gender), body mass index, Padua Prediction score, comorbidities and/or known risk factors for VTE such as myocardial infarction, hypertension, diabetes mellitus (DM), rheumatological diseases, congestive heart failure, peripheral vascular disease, connective tissue disorders, peptic ulcer disease, chronic kidney disease, lymphoma, liver disease, cancer, Acquired immunodeficiency syndrome (AIDS) and smoking. Data regarding the severity of COVID-19 (asymptomatic, mild, moderate, severe), thromboprophylaxis, hospital stay, intensive care unit (ICU) admission, ICU length of stay, mechanical ventilation, ventilatory days, and mortality were also collected. Data on anticoagulation therapy, such as Enoxaparin, Dalteparin, Rivaroxaban, Dabigatran, Apixaban, Heparin, and Aspirin, were collected.

The primary outcome was defined as TEE occurrence, which was diagnosed by clinical presentation (physicians and nursing notes), laboratory investigations, and/or radiologic findings. The type of TEE included deep vein thrombosis (DVT) of the limbs, pulmonary embolism (PE), AMI (type I and II AMI), stroke and thrombosis at other sites, including femoral artery ischemia, portal vein thrombosis, hepatic vein thrombosis, Internal jugular vein thrombosis, cephalic vein thrombosis, circumferential mural thrombosis, splenic artery embolism and subclavian artery thrombosis. The AMI was diagnosed by clinical symptoms, electrocardiogram (ECG) changes (ST-segment elevation MI (STEMI) or Non-ST-segment elevation MI (NSTEMI), and elevated high-sensitive troponin T levels above 14 ng/mL. PE was detected by Computed Tomography pulmonary angiography (CTPA) as indicated for deteriorating respiratory status. Clinical findings and duplex ultrasonography confirmed DVTs and arterial thromboses, while stroke was diagnosed based on clinical presentation and computer tomographic (CT) imaging. Padua Prediction Score is a tool for VTE risk assessment based on 11 criteria (a score of 4 and above indicates higher risk and a need for prophylactic measures).^21^

**Figure 1.**
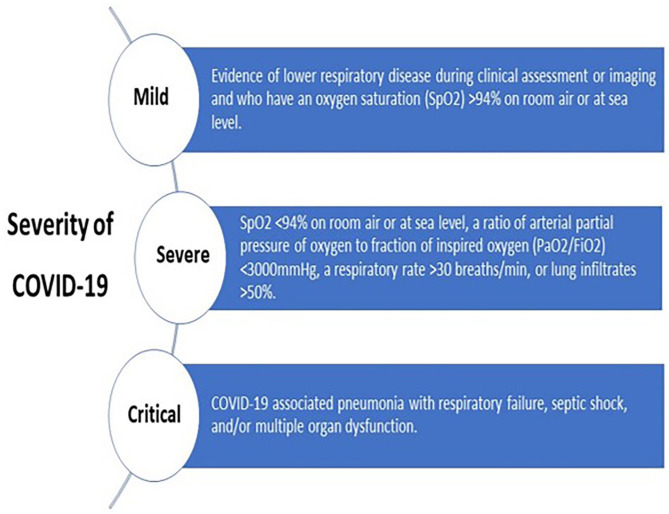
Severity of COVID-19 disease.

**Figure 2.**
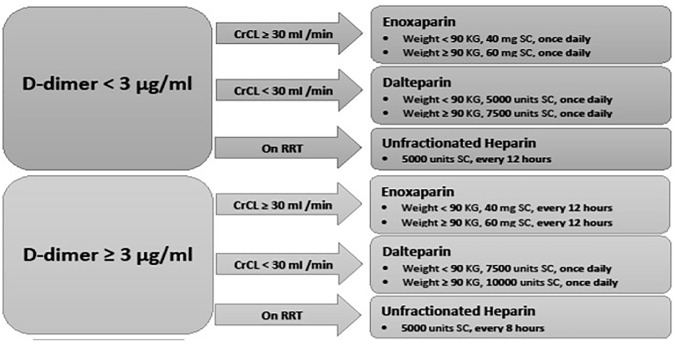
COVID-19 thromboprophylaxis protocol during the first wave in Qatar.

SARS-CoV-2 infection was confirmed by reverse transcription polymerase chain reaction (RT-PCR) performed on the nose/throat swab samples, which could be either asymptomatic or symptomatic. Symptomatic COVID-19 patients were classified as mild, severe, and critically ill according to the HMC-Communicable Disease Center (CDC) protocols (Figure 1). Thromboprophylaxis was the standard of care for all COVID-19 patients, and the institutional protocol for thromboprophylaxis is outlined in Figure 2.

Ethical approval for this study was granted from the Research Ethics Committee, Medical Research Center, Hamad Medical Corporation, Doha, Qatar (MRC-01-20-1047), waived the need for informed consent for this retrospective descriptive study, allowing the inclusion of all hospitalized COVID-19 patients.

### Statistical analysis

Data were expressed as numbers, percentages, and mean ± standard deviation or medians with range whenever appropriate. Patients were categorized based on the history of prior MI, age (<55 vs ⩾55), and gender. Categorial variables were compared using the Chi-square test. A multivariable regression analysis was performed for predictors of mortality after adjustment for relevant variables such as age, gender, DM, prior MI, new AMI, procalcitonin, CRP, ferritin, and fibrinogen. Data were expressed as odds ratio and 95% confidence intervals. A two-tailed *P* value <.05 was considered for statistically significant difference. Data analysis was carried out using the Statistical Package for Social Sciences version 21 (SPSS Inc., Chicago, IL).

## Results

In this nation wide-based study over the period of 7 months, a total of 16,903 patients with confirmed COVID-19 were admitted to various COVID-19 treatment facilities, of which 210 (1.2%) developed TEE (MI = 76.2%; DVT = 11%; stroke = 3.8%; PE = 1.9% and others = 7.1%). COVID-19 was severe in 32% of cases. AMI occurred in 160 patients in terms of 42.5 % STEMI and 57.5% NSTEMI. The other TEE included femoral artery ischemia (n = 2), portal vein thrombosis (n = 5), hepatic vein thrombosis (n = 1), Internal jugular vein thrombosis (n = 2), cephalic vein thrombosis (n = 1), circumferential mural thrombosis (n = 1), splenic artery embolism (n = 1), subclavian artery thrombosis (n = 1). The majority were males (89%), 55% were <55 years old, 72% were South Asians, and 80.5% had prior MI (Table 1). The mean body mass index was 26.6 ± 5.4, and the Padua prediction score was 4.02 ± 1.78. Hypertension (64.3%) and diabetes mellitus (60.5%) were the other frequent comorbidities after AMI. Although two-thirds of the cases had mild COVID-19 disease, almost 60% of the cases required ICU admission because of other complications, including TEE, and 27% required mechanical ventilation during hospitalization. Most patients (85%) received thromboprophylaxis per the standard institutional protocol.

**Table 1.** Clinical characteristics, comorbidities, management, and outcomes of patients with COVID-19 and developed thromboembolism events (n = 210).

Variable	Value	Variable	Value
Age in years	54.1 ± 13.4	*Severity of COVID*	
Females	23 (11.0%)	Mild	142 (68.3%)
Males	187 (89.0%)	Severe	32 (15.4%)
Body mass index	26.6 ± 5.4	Critical	34 (16.3%)
Padua prediction score	4.02 ± 1.78	*Treatment*	
*Comorbidities*		Enoxaparin	186 (88.6%)
Old myocardial infarction	169 (80.5%)	Dalteparin	25 (11.9%)
Hypertension	135 (64.3%)	Rivaroxaban	11 (5.2%)
Diabetes mellitus	127 (60.5%)	Dabigatran	5 (2.4%)
Rheumatological diseases	3 (1.4%)	Apixaban	2 (1.0%)
Congestive heart failure	15 (7.1%)	Heparin	127 (60.5%)
Peripheral vascular disease	2 (1.0%)	Aspirin	168 (80.0%)
Connective tissue disorders	1 (0.5%)	No-VTE prophylaxis	32 (15.2%)
Peptic ulcer disease	5 (2.4%)	VTE prophylaxis	178 (84.8%)
Chronic kidney disease	31 (14.8%)	Hospital length of stay (d)	17.1 (0.4-238)
Lymphoma	1 (0.5%)	ICU admission	123 (59.7%)
Liver disease	2 (1.0%)	ICU length of stay (d)	4.5 (0.2-89.7)
Cancer	11 (5.2%)	Mechanical ventilation	58 (27.6%)
AIDS	1 (0.5%)	Ventilatory days	1 (1-49)
Smokers	40 (19.0%)	Mortality	34 (16.2%)

**Figure 3.**
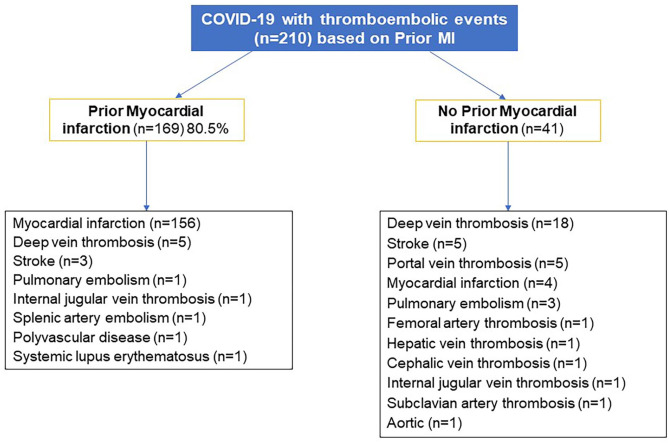
COVID-19 cases with thromboembolic events (n = 210) based on the presence of a prior MI.

The overall mortality rate was 16.2%. Figure 3 shows the distribution of TEE in COVID-19 patients based on prior history of AMI. Among patients with a history of MI (n = 169), 92.3% had developed another AMI. On the other hand, DVT (44%) and portal vein thrombosis (12.2%) occurred more frequently than new AMI (9.8%) in non-prior AMI patients. Table 2 compares those who had non-prior AMI versus prior AMI. Patients with a prior AMI were more male, diabetics, had mild to moderate COVID-19 disease and had a new AMI. Patients with no prior AMI had more severe COVID-19 disease, stayed longer in the ICU, had higher initial and peak serum D-Dimer, ferritin, and procalcitonin, and higher rates of DVT, PE, stroke, and in-hospital mortality.

**Table 2.** Comparison between COVID-19 patients who had new thromboembolism events without having prior MI and those with prior MI.

Variable	Non-prior MI (N = 41)	Prior MI (N = 169)	*P* value
Age in years	52 (40-63)	53 (45-62)	.42
Male gender	80%	91%	.050
Diabetes mellitus	46%	64%%	.03
Hypertension	56%	66%	.22
Chronic kidney disease	17%	14%	.64
*COVID-19 severity*			.001 for all
Mild	58.5%	70.7%	
Severe	4.9%	18%	
Critical	36.6%	11.4%	
Left ventricular ejection fraction	46 (37-55)	51(39-57)	.58
*ECG findings in 160 AMI patients*			—
STEMI (n = 90)	1	89	
NSTEMI (n = 70)	3	67	
DVT prophylaxis	92.7%	82.8%	.11
ICU length of stay (d)	20 (6-50)	3.5 (1.8-10.8)	.001
Initial D-Dimer (0.0-0.44 mg/L)	1.30 (0.67-2.80)	0.61 (0.30-1.50)	.001
Peak D-Dimer (0.0-0.44 mg/L)	4.47 (1.58-25.4)	0.88 (0.45-4.96)	.001
Initial ferritin (30-553 ug/L)	523 (349-1296)	290 (182-649)	.003
Peak ferritin (30-553 ug/L)	1209 (485-5087)	442 (233-939)	.003
Initial interleukin-6 (⩽7 pg/mL)	62 (12-92)	35 (17-156)	.94
Peak interleukin-6 (⩽7 pg/mL)	203 (42-1881)	95 (20-393)	.18
Initial procalcitonin* (ng/mL)	0.74 (0.02-87.3)	0.11 (0.02-100)	.003
Peak procalcitonin	2.05 (0.02-100)	0.19 (0.02-100)	.008
Initial C-reactive protein (⩽5 mg/L)	88 (18-226)	17 (5-72)	.002
Peak C-reactive protein	214 (46-311)	47 (10-171)	.002
Serum troponin (⩽14 ng/L)	8.5 (5.3-39)	73 (23-244)	.001
*Type of TEE*; N (%)			.001 for all
Deep vein thrombosis	18 (43.9%)	5 (3.0%)	
Pulmonary embolism	3 (7.3%)	1 (0.6%)	
Acute myocardial infarction	4 (9.8%)	156 (92.3%)	
Stroke	5 (12.2%)	3 (1.8%)	
Others	11 (26.8%)	4 (2.4%)	
Mortality (N = 34)	26.8% (N = 11)	13.6% (N = 23)	.039

Abbreviations: N, number of patients; TEE, thromboembolic event.

Continuous variables are given as Median and interquartile range.

* <0.5 ng/mL represents a low risk of severe sepsis and/or septic shock >2.0 ng/mL represents a high risk of severe sepsis and/or septic shock.

**Figure 4.**
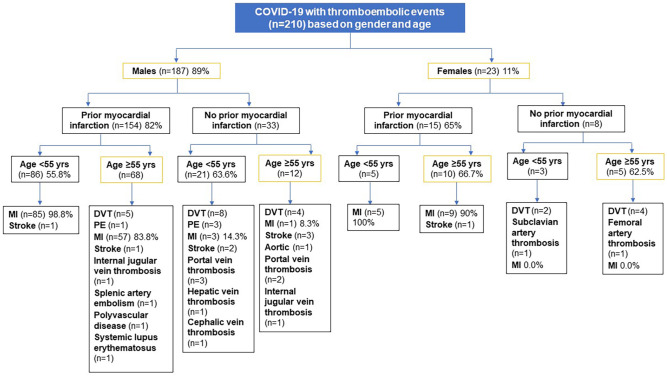
COVID-19 cases with thromboembolic events (n = 210) based on gender and age.

Figure 4 compares the COVID-19-related TEE cases based on demographics and prior history of MI. The occurrence of new MI (98.8%) was more evident among young (<55 years) male patients who had prior MI; however, it was also reported more in young males without prior MI (14.3%) as opposed to the older age group. Both young and older females were more likely to have a new MI with a history of MI. Furthermore, regardless of age groups, there were no occurrences of new MI in females who did not have a prior history of MI. MI was reported in 74.2% of those who received VTE prophylaxis versus 87.5% of those who did not receive VTE prophylaxis (*P* = .001). The common causes of death in patients with TEE included respiratory failure, septic shock, cardiogenic shock, and malignancy.

Table 3 compares the COVID-19 patients based on the type of acute myocardial infarction. It showed that NSTEMI was predominant and mostly associated with severe COVID-19 status. Patients with STEMI were all males and had less severe COVID-19 and a higher number of the affected coronary vessels.

**Table 3.** Patients with COVID-19 based on the type of acute myocardial infarction (N = 160).

Variable	NSTEMI	STEMI	*P* value
Number (%)	92 (57.5)	68 (42.5)	
Age in years	54.7 ± 15	53.9 ± 13	.72
Male gender	84.8%	100%	.001
Prior MI	96.7%	98.5%	.47
Mild COVID-19	67.4%	79.4%	.09
Severe-critical COVID-19	32.6%	20.6%	.09
Coronary angiography done (n = 92)	57.1%	21.2%	.001
Single-vessel disease	12.1%	37.9%	.001
Two-vessel disease	11%	18.2%	.001
Three-vessel disease	19.8%	22.7%	.001
Percutaneous coronary intervention	5.5%	16.7%	.02
Mortality	14.1%	11.2%	.66

### Mild versus severe-critical COVID-19

Patients with mild COVID-19 had less mortality (5.6% vs 39.4%), higher rates of prior MI (83% vs 74%), hypertension (60% vs 40%), and STEMI (46.5% vs 31.8%) and fewer rates of chronic kidney disease (9.2% vs 27.3%), chronic heart failure (3% vs 17%), and NSTEMI (53.5% vs 68.2%) compared to those who had severer COVID-19. DM rate was comparable between the groups.

### Predictors of mortality among COVID-19 patients who had TEE

Table 4 shows multivariable logistic regression analysis for predictors of mortality and finds that severity of COVID-19 (severe or critical type) was the independent predictor of mortality (OR 10; 95% CI 1.617-67.194) after adjustment for age, gender, DM, CRP, Ferritin, Procalcitonin, fibrinogen values, and prior or new MI.

**Table 4.** Predictors of mortality in patients with COVID-19 and MI.

Variable	*P* value	Odds ratio	95% confidence interval	
Gender	.979	1.031	0.109	9.797
Age	.226	1.028	0.983	1.076
Diabetes mellitus	.266	0.430	0.097	1.899
C-Reactive protein	.821	1.001	0.992	1.010
Serum ferritin	.421	1.000	1.000	1.000
Serum fibrinogen	.390	0.849	0.585	1.232
Serum procalcitonin	.910	0.998	0.962	1.036
Prior AMI	.979	0.970	0.094	10.009
Severe COVID-19	.014	10.424	1.617	67.194
New AMI	.972	0.961	0.104	8.896

## Discussion

Although the occurrence of AMI in COVID-19 patients has been addressed in several studies, the present study uniquely describes the association between a new MI in COVID-19 patients with a prior MI. Patients with prior MI developed new MI in almost 80% of cases in this study. However, the mortality was higher in patients without a history of MI, which could be due to the severity of COVID-19 and/or the development of PE or stroke. The laboratory markers such as D-Dimer, ferritin, and procalcitonin were greater in those with no prior MI. The severity of COVID-19 was an independent predictor of mortality in our study after adjustment for age, gender, DM, prior or new AMI, CRP, Ferritin, Procalcitonin, and fibrinogen values. In the present study, the severe forms of COVID-19 were associated with higher rates of new NSTEMI and mortality than the milder COVID-19. Whereas mild COVID-19 was associated with higher prior MI and new STEMI rates. The literature reports a wide range of incidences of thrombotic complications associated with COVID-19 disease, with rates varying between 3% and 85%.^2,3^ Hypercoagulability has been recognized as a critical determinant of prognosis in COVID-19 patients. The reported thrombotic complications included PE, DVT, ischemic stroke, MI, and/or systemic arterial embolism.^22^ A study conducted in the Netherlands found that among hospitalized COVID-19 patients receiving prophylactic anticoagulation, there was a cumulative incidence of thrombotic complications reaching 49%.^2^ A similar study by Poissy et al^5^ reported thromboembolic complications could occur in one to five of COVID-19 patients admitted to the ICU.

In contrast to these studies, our research revealed an exceptionally lower incidence of 1.2% for all thromboembolic complications in our cohort. One potential explanation for the marked difference could be our consistent use of a single, stringent thromboprophylaxis protocol across all treatment facilities, coupled with dose adjustments based on daily D-Dimer level measurements. An additional factor that could account for this variation is the absence of routine TEE screening, as TEE examinations were solely conducted upon clinical suspicion, potentially leading to the possibility of missed diagnosis of TEE in some asymptomatic patients. However, studies that adopted mandatory TEE screening, particularly for ICU patients, would be expected to find higher incidences of TEE.^4^ In a study from China of 81 ICU patients not receiving routine thromboprophylaxis, the incidence of VTE was 25%.^7^

Although the exact pathophysiology of COVID-19 remains unexplored, the current understanding suggests an intricate relationship between the immune and coagulation systems, resulting in a prothrombotic state.^23^ Several mechanisms have been proposed for developing thrombotic microangiopathy and TEE.^1,24,25^ Direct endothelial injury followed by a hyperinflammatory response may have a role in culminating a unique coagulopathic effect, distinct from other coagulopathies in critically ill patients.^1,14^ Moreover, an increase in proinflammatory cytokines, especially IL-6, stimulates tissue factor release by mononuclear cells, resulting in thrombin generation, which compounds the systemic coagulopathic process.^1^ Studies based on autopsies have shown that lung injury in COVID-19 involves not just the interstitial tissue, as previously believed, but also affects the endothelium.^25,26^ These studies demonstrated the presence of platelet–fibrin thrombi, which supports the notion that SARS-COV2 promotes a thrombogenic response by direct and cytokine-mediated endothelial injury and platelet and fibrin complex deposition.^24-27^

In our study, the most frequent thrombotic complication was AMI (76.2%), followed by DVT (11%), stroke (3.8%), and PE (1.9%). Our findings are in contrast with an earlier report, which showed a lower incidence of AMI and ischemic stroke (1.2%) and VTE (2.7%) among hospitalized COVID-19 patients.^28^ However, the overall incidence of acute thrombotic events was relatively higher (5.2%) as compared to our cohort. Despite the low overall incidence of TEEs in our study, the interesting finding was the higher rate of AMI (76%) in patients with COVID-19, especially those with a prior history of MI. These findings are similar to those of a large study involving 86,742 COVID-19 patients and 348,481 matched controls from Sweden. The study found that the risk of AMI and ischemic stroke was significantly higher within the first 2 weeks following COVID-19.^17^ This suggests an elevated risk of cardiovascular events shortly after COVID-19. Another study showed an incidence of AMI to be 15% in COVID-19 patients prospectively followed from the diagnosis.^29^ Prior history of AMI may predispose patients with COVID-19 to develop an AMI, possibly because the SARS-CoV-2 infection directly affects the cardio-vasculature of the patients.^30^ It has been reported that having a preexisting cardiovascular disease is a significant risk factor for experiencing a severe clinical course of COVID-19 and is linked to unfavorable outcomes.^31^ A recent meta-analysis of four studies encompassing a population of over one million individuals showed that individuals who recovered from COVID-19 had a 0.5% increased risk of AMI during an 8.5-month follow-up.^18^ However, the analysis did not focus on MI occurrences during the acute phase of COVID-19. Notably, the underlying pathophysiology of MI is diverse and multifaceted, involving inflammation, endothelial dysfunction, platelet activation, and psychosocial stressors.^30,31^ Therefore, COVID-19 can influence the risk of AMI through a complex interplay of various factors. Acute viral infection may result in an increased inflammatory response and may have a role in triggering acute coronary syndrome. The resultant endothelial damage may be compounded by underlying atherosclerosis or even an already diseased myocardium that may trigger a new onset MI.^32^ In general, before the era of COVID-19, studies demonstrated that more than 20% of patients with AMI had a history of a prior MI.^33,34^

Interestingly, in our study, venous thromboembolism occurred more frequently in COVID-19 patients without a history of AMI. This could be related to the hypercoagulable state induced by the virus, endothelial damage, systemic inflammation, immobility during hospitalization, and various other risk factors that collectively contribute to a higher risk of VTE in COVID-19 patients, regardless of their cardiovascular history.^35^ Further to support these findings, nearly 20% of patients in our cohort had non-MI thromboembolic events that had comorbidities such as malignancy at the time of the COVID-19, history of significant TEE, and DVT. This raises the question of whether COVID-19 truly creates an increased risk of non-MI TEE compared to other disease processes.

The literature reported variation in the rate of occurrence of AMI between men and women in different age groups during the pandemic.^36-39^ In our cohort, the occurrence of new AMI was more evident among young (<55 years) males with or without a prior AMI than in the older group. Furthermore, regardless of age, females were more likely to have new AMI who had a history of old AMI. The possible explanation is that COVID-19 may trigger or accelerate the presentation of preexisting coronary artery disease, even in younger adults. Our findings are in line with a previous review of AMI in COVID-19 cases, which identified a higher rate of AMI among males (81.1%) than females (18.9%), and the average age of the patients was 52.8 ± 15.6 years.^40^

In contrast to our findings, a Swedish study on patients who experienced AMI during the pandemic found no disparities in terms of age, gender, and comorbidities, except for lower occurrences of a history of AMI and coronary artery bypass grafting.^37^ Similarly, another study on AMI during COVID-19 reported a lower likelihood of patients with a prior history of coronary artery disease in comparison to those who presented before the pandemic.^39^ The NACMI Registry demonstrated that 26.3% of those who had AMI and COVID-19 were females, and mostly, they had STEMI without an identified culprit lesion than men (33% vs 18%). However, the in-hospital mortality (≈30%) was comparable in both genders.^41^ In our study, data for patients with no prior AMI showed that males had severe COVID-19 (45.5% vs 12.5%) and were more smokers (12% vs 0.0%) than females, while both genders were comparable for age, DM, hypertension and chronic kidney disease.

Overall, the potential mechanisms of AMI in COVID-19 may include cytokine storm, adrenergic stimulation, coagulopathy, ACE-2 mediated effect, hypotension, hypoxia, production of neutrophil extracellular traps (NETs) from intraplaque and circulating neutrophils, increased platelet activity, impaired fibrinolysis and decreased anticoagulant function of the endothelium.^18,42^ Prior data showed a significant drop in the number of STEMI patients invasively treated from 2019 to 2020, with an 18.9% reduction in admissions for STEMI.^43^ Patients treated in 2020 also had longer ischemia and door-to-balloon time. The number of primary PCI dropped by more than half in the first 3 months of 2020 compared to 2018 and 2019, and the number of patients treated with fibrinolysis increased by 2 to 3 times. A longer time to reperfusion was reported among patients treated in 2020.^43,44^ During the pandemic surges, a prior study reported that the number of patients presenting with STEMI declined substantially, Hospital reperfusion strategies were modified, and delays in reperfusion were observed around the world. Poorer STEMI-related outcomes, including higher rates of in-hospital mortality.^45^ Moreover, in infected and recovered patients, a persistent hypercoagulable state increases the risk of coronary thrombosis at the sites of plaque disruption in COVID-19 patients.^18^ Moreover, a case report indicated that there is a substantial need to assess patients with mild COVID-19 symptoms who may be at risk of developing cardiovascular events and to consider a modification of the current cardiovascular risk factors to include COVID-19 as a new risk factor.^46^

### Limitations

It is worth considering certain limitations when interpreting the present study’s findings. First, the retrospective nature of this study comes with inherent limitations, particularly related to missing information, which lowers the overall quality of evidence. Although the present analysis could encompass the entire population of the country due to the centralized healthcare system, there is still the possibility that patients admitted to other healthcare facilities might have yet to be included. Another limitation is that all TEE investigations were performed solely on clinical suspicion (not a primary outcome). Therefore, this approach may have missed clinically insignificant TEE, which was not confirmed and overlooked without specific on-time investigation. An additional limitation worth acknowledging is the absence of a control group who have no history of MI to effectively evaluate the influence of the COVID-19 pandemic on individuals with a prior history of MI. Finally, some of our findings may be difficult to explain by unmeasured confounding factors, as there is a significant overlap between the risk factors for vascular disease and COVID-19.

Moreover, the timing between the old and new MI is not documented. The current study did not capture the long-term outcomes in such a population. A recent systematic review, including five studies, evaluated the long-term consequences of COVID-19 survivors following AMI. It showed an increased risk of cardiac events in COVID-19 survivors than in the general population, with poorer long-term outcomes.^47^

## Conclusions

This study suggests that the rate of TEE, particularly AMI, increases during COVID-19 in patients who had a prior MI. Moreover, age and gender greatly impacted the occurrence of TEE. Increased attention should be given to patients who have thrombotic risk factors during such viral infection. New MI is more evident in young male patients with or without prior MI after COVID-19; however, females were more likely to have new MI in those who had a history of old MI.
